# SGLT2 inhibitors therapy protects glucotoxicity-induced β-cell failure in a mouse model of human K_ATP_-induced diabetes through mitigation of oxidative and ER stress

**DOI:** 10.1371/journal.pone.0258054

**Published:** 2022-02-18

**Authors:** Zeenat A. Shyr, Zihan Yan, Alessandro Ustione, Erin M. Egan, Maria S. Remedi

**Affiliations:** 1 Division of Endocrinology, Metabolism and Lipid Research, Department of Medicine, Washington University in St. Louis, St. Louis, Missouri, United States of America; 2 Center for the Investigation of Membrane Excitability Diseases, Washington University in St. Louis, St. Louis, Missouri, United States of America; 3 Department of Cell Biology and Physiology, Washington University in St. Louis, St. Louis, Missouri, United States of America; University of Michigan, UNITED STATES

## Abstract

Progressive loss of pancreatic β-cell functional mass and anti-diabetic drug responsivity are classic findings in diabetes, frequently attributed to compensatory insulin hypersecretion and β-cell exhaustion. However, loss of β-cell mass and identity still occurs in mouse models of human K_ATP_-gain-of-function induced Neonatal Diabetes Mellitus (NDM), in the absence of insulin secretion. Here we studied the temporal progression and mechanisms underlying glucotoxicity-induced loss of functional β-cell mass in NDM mice, and the effects of sodium-glucose transporter 2 inhibitors (SGLT2i) therapy. Upon tamoxifen induction of transgene expression, NDM mice rapidly developed severe diabetes followed by an unexpected loss of insulin content, decreased proinsulin processing and increased proinsulin at 2-weeks of diabetes. These early events were accompanied by a marked increase in β-cell oxidative and ER stress, without changes in islet cell identity. Strikingly, treatment with the SGLT2 inhibitor dapagliflozin restored insulin content, decreased proinsulin:insulin ratio and reduced oxidative and ER stress. However, despite reduction of blood glucose, dapagliflozin therapy was ineffective in restoring β-cell function in NDM mice when it was initiated at >40 days of diabetes, when loss of β-cell mass and identity had already occurred. Our data from mouse models demonstrate that: *i)* hyperglycemia *per se*, and not insulin hypersecretion, drives β-cell failure in diabetes, *ii)* recovery of β-cell function by SGLT2 inhibitors is potentially through reduction of oxidative and ER stress, *iii)* SGLT2 inhibitors revert/prevent β-cell failure when used in early stages of diabetes, but not when loss of β-cell mass/identity already occurred, *iv)* common execution pathways may underlie loss and recovery of β-cell function in different forms of diabetes. These results may have important clinical implications for optimal therapeutic interventions in individuals with diabetes, particularly for those with long-standing diabetes.

## 1. Introduction

Reduced pancreatic β-cell function and mass contribute to both type 1 diabetes (T1D) and type 2 diabetes (T2D) [1]. Reduction of β-cell function is considered an early event, while loss of β-cell mass occurs closer to clinical manifestation [2–5]. Chronic high glucose induce β-cell membrane hyperexcitability, persistently elevated intracellular calcium concentration and insulin hypersecretion, all critically contributing to loss of β-cell mass in diabetes [6–8]. However, loss of β-cell mass still occurs in K_ATP_-gain-of-function (K_ATP_-GOF) mouse model of human neonatal diabetes mellitus (NDM), in which all of these factors are absent due to K_ATP_ overactivity in pancreatic β-cells [9–13]. Chronic hyperglycemia can also induce β-cell overstimulation, oxidative and endoplasmic reticulum (ER) stress leading to β-cell exhaustion, and loss of β-cell mass and identity [4, 7, 14–16]. However, most of the studies have been performed in human pancreases from T2D individuals and in animal models of T2D and obesity, and little is known about mechanisms underlying and temporal progression of loss of functional β-cell mass in monogenic diabetes, in the absence of compensatory increase in insulin secretion.

Loss of functional β-cell mass in diabetes seems to be independent of the antidiabetic therapy, with most antidiabetic agents initially effective as monotherapy, but failing to reduce blood glucose over time [17–19], requiring add-on combinational therapies. Sodium glucose transporter 2 inhibitors (SGLT2i) are a new class of antidiabetic drugs that inhibit glucose reabsorption in the kidneys and increase glucose excretion in the urine. Because their mechanism of action is independent of insulin secretion or action, SGLT2i can be used in combination with other therapies [20]. T2D individuals treated with SGLT2i demonstrate improved glycemic control, increased glucose- and incretin-stimulated insulin secretion and enhanced insulin sensitivity as well as reduced blood pressure, decreased plasma lipids and reduced risk for cardiovascular events [21–24]. *Db/db* and *ob/ob* mouse models of T2D and obesity treated with SGLT2i (luseogliflozin, ipragliflozin or dapagliflozin) demonstrate reduced blood glucose, augmented insulin and GLP-1 secretion, increased β-cell mass, β-cell replication and α- to β-cell conversion, and restored β-cell identity [25–30]. Moreover, *db/db* mice with SGLT2 deletion show improved insulin sensitivity, increased β-cell proliferation and decreased β-cell death [31]. Streptozotocin-induced diabetic mice treated with empagliflozin demonstrate increased insulin mRNA, serum insulin, and β-cell area and proliferation [32]. Although improved β-cell function by SGLT2i has been suggested in humans and rodents, the underlying mechanisms and timeframe of this effect remain elusive, with most studies performed in the setting of obesity and T2D. Here we determined the underlying mechanisms and temporal progression to β-cell failure and established the effects of SGLT2i therapy in an insulin secretory-deficient mouse model of K_ATP_-GOF induced NDM.

## 2. Materials and methods

### Mouse models

Neonatal diabetic mice were generated as previously described [9]. Briefly, mice expressing Kir6.2 (K185Q,ΔN30) mutant transgene under the Rosa26 locus promoter were crossed with tamoxifen-inducible Pdx-Cre mice, to generate transgenic mice expressing the NDM mutation in β-cells. To induce transgene expression, 12 to14-week old mice were injected with five consecutive daily doses of tamoxifen (50 μg/g body weight). Littermate single transgenic (Rosa-kir6.2, Pdx-cre) were also injected with tamoxifen and used as controls since to date, no significant differences in phenotype were found. Both male and female mice were used for all experiments as no significant sex-based differences were found. Mice were maintained on a 12-hour light/dark cycle. Animals were housed in BJCIH animal facility with a 24hs surveillance with veterinarian and personnel assigned to the facility. Power analysis determined the sample size, and it is stated in each experimental design and figures. Experimental groups were randomized, and treatment separation was assigned.

### Ethics statement

This study was carried out in strict accordance with the recommendations in the Guide for the Care and Use of Laboratory Animals of the National Institutes of Health. The protocol was approved by the Washington University School of Medicine Animal Care and Use Committee (IACUC, Protocol Number: 19–1078). Tissue collection was performed at the end of the experiments at a predetermined time, with the animals euthanized by inhalation of Isoflurane in a chamber, and then cervical dislocation. All efforts were made to minimize suffering.

### Plasma hormones, and blood and urine glucose measurements

Blood glucose was measured either randomly or after an overnight fast as indicated by using the one-touch Bayer Contour T5 glucometer (Mishawaka, IN). Plasma insulin and glucagon levels were quantified using a rat/mouse insulin and mouse glucagon ELISA (Crystal Chem, IL). Urine glucose was analyzed by glucose-Autokit (Wako Diagnostics, Richmond, VA). Plasma lipids were measured by Washington University Diabetes Research Center Metabolic Tissue Function Core (http://diabetesresearchcenter.dom.wustl.edu/diabetes-models-phenotyping-core/). For all *in vitro* analyses, at least three independent animals were used. Cells and tissues from each animal were kept separated and analyzed individually. Data collection was stopped at predetermined, arbitrary time as 10 days after initiation of DAPA/vehicle treatment. No data were excluded. Further methods details are available in the following sections or in the Supplementary Materials section.

### Glucose Tolerance Tests (GTT) and Insulin Tolerance Tests (ITT)

GTTs and ITTs were performed after overnight or six-hour fast, respectively. Blood glucose was measured before (time 0) and after (15, 30, 45, 60, 90 and 120 min) intraperitoneal (ip) injection of 1.5 mg/kg dextrose (GTT) or 0.5 U/kg human insulin (ITT, HI-210 Lilly).

### Pancreatic islet isolation

For islet isolation, euthanized mice were perfused through the bile duct with Hank’s solution containing collagenase type XI (Sigma, St. Louis, MO). Pancreases were removed, digested for 11 minutes at 37°C in a water bath, hand shaken, and washed in cold Hank’s solution. Islets were handpicked under a stereo microscope in RPMI (11mM glucose) media (ThermoFisher Scientific) supplemented with fetal calf serum (10%), penicillin (100 U/ml), and streptomycin (100 μg/ml). For all assays involving islets, freshly isolated islets were immediately processed and frozen at -80C to avoid changes induced in the absence of DAPA.

### Insulin and proinsulin content measurement

Total insulin and proinsulin content were measured using rat/mouse insulin ELISA kit (Crystal Chem, IL) and proinsulin ELISA kit (Mercodia, Uppsala, Sweden) respectively from batches of ten size-matched islets after acid-ethanol extraction [12].

### Quantitative PCR analysis

Islet RNA extraction and qPCR analysis was performed as described previously [10]. Primers used to determine ER stress markers [33] and mature islet cell markers [10] have been published.

### Western blot analysis

15μg of total protein lysate was loaded per lane. Blots were incubated overnight with the following antibodies: Beta-actin (1:1000; EMD Millipore, MO cat #MAB1501R), Proinsulin (1:1000; CST, MA, cat #3014S), TXNIP (1:1000; MBL International, MA, cat #K0205-3), sXBP1 (1:1000; Santa Cruz Biotechnology, TX, cat #sc-7160), BiP (1:1000, CST cat #3183), Prohormone convertase 1/3 (1:1000; CST, MA, cat #11914), Prohormone convertase 2 (1:1000; CST, MA, cat #14013T), SERCA2b (1:1000, Santa Cruz Biotechnology, TX, cat #SC-8095). Blots were washed and probed with RDye infrared fluorescent dye-labeled secondary antibody conjugates (1:10,000; LI-COR biotechnology). Fluorescence intensity was determined by image studio Lite (LI-COR biotechnology). Raw uncropped western blot images can be found at https://figshare.com/search?q=10.6084%2Fm9.figshare.17161070.

### Immunohistochemical and morphometric analysis

Pancreases from control and NDM mice were fixed in 10% NBF, and paraffin-embedded after serial dehydration for sectioning. Four- to eight mice from each genotype were sampled on 5μm thick sections, 25μm apart (spanning the whole pancreas) and used for immunohistochemical/morphometric analysis. For morphometric analysis, at least 3 pancreatic sections from 3–8 mice from each genotype were covered systematically by accumulating images from non-overlapping fields on an inverted EXC-500 fluorescent microscope (Visual Dynamix, Chesterfield, MO). Hematoxylin-Eosin (HE) staining was carried out as described previously [10]. Briefly, slides were stained with rabbit anti-insulin (1:100 CST cat#C27C9) and mouse anti-glucagon (1:100, Abcam cat#ab10988) antibodies and their distribution visualized using secondary antibodies conjugated with AlexaTM 488 or AlexaTM 594 (Molecular Probes, Eugene-OR) using an EXC-500 fluorescent microscope (Visual Dynamix, Chesterfield, MO). Whole pancreatic area and islet area were determined by H&E staining, and β-cell area and intensity by insulin staining (as above). Β-cell mass was calculated as the product of relative β-cell area to whole pancreatic area and pancreatic weight for each mouse.

### Reactive oxygen species

Islets were dispersed into single cells using Accutase (Sigma-Aldrich). They were plated in RPMI complete media overnight on ploy-D-lysine (Sigma-Aldrich) coated glass-bottom culture dishes (MatTek Corp). After overnight culture, cells were incubated in KRB containing 2.8 mM glucose for one hour, followed by 16.7 mM glucose for one hour at 37°C. In the last 30 minutes of incubation, CellROX Deep Red Reagent (final concentration of 5μM, Molecular Probes ThermoFisher Scientific, MA) and live nuclear reagent (Hoeschst 33342, Molecular Probes) were added. After 30 min, cells were washed with PBS 3 times and imaged using a Leica DMI 4000B inverted microscope (Lecia microsystems, IL). Fluorescence intensity was quantified by image studio Lite (LI-COR biotechnology).

### Calcium measurements

The genetically encoded calcium sensor pRSET-RcaMP1h was a gift from Loren Looger (Addgene plasmid # 42874). The coding sequence of the RcaMP1h sensor was subcloned into the pShuttle vector, and recombinant adenovirus particles were produced following the AdEasy XL Adenoviral Vector System protocol (#240010 Agilent Technologies). Islets were transduced using a microfluidic device to obtain uniform infection of the islet cells throughout the islet volume and were incubated overnight before imaging. Imaging on islets was performed on the LSM880 inverted microscope (Zeiss Inc), using a heated stage-top incubator (Pecon GmbH) 24 hours post transduction. Islets were imaged in KRBH medium (NaCl 128.8 mM, KCl 4.8 mM, KH2PO4 1.2 mM, MgSO4 1.2 mM, CaCl2 2.5 mM, NaHCO3 5mM, HEPES 10 mM, pH 7.40, 0.1% BSA) supplemented with the desired glucose concentration. RcaMP1h fluorescence was excited with a 561 nm laser, and the emission was collected through a 570–650 nm bandpass.

### Dapagliflozin treatment

NDM, db/db and ob/ob mice were randomly separated into two treatment groups–vehicle or–Dapagliflozin (DAPA, SelleckChem, Houston, TX), a sodium-glucose transporter 2 inhibitor. DAPA was suspended in vehicle containing 30% Polyethylene glycol, 5% Propylene glycol, 0.4% tween-80 as recommended by the manufacturer. Twelve to fourteen- week-old NDM mice were injected with 5 consecutive doses of tamoxifen to induce diabetes. Mouse groups were assigned randomly, and the study was not blinded. On day 7 after tamoxifen injection, NDM mice received a daily oral gavage of either vehicle or DAPA (10 mg/kg of body weight) for 10 days. Eleven-week-old *db/db* and *ob/ob* mice also received a daily oral gavage of either vehicle or DAPA (10 mg/kg of body weight) for 10 days. Vehicle and DAPA treated mice were monitored through blood glucose measurements daily and blood serum collection and then euthanized for *ex vivo* analysis. Data collection was stopped at predetermined, arbitrary time as 10 days after initiation of DAPA/vehicle treatment. No data were excluded. Further methods details are available in the following sections or in the Supplementary Materials section.

### Statistical analysis

Data are presented as mean ± standard deviation of the mean. Statistical differences between two groups were determined using Student’s t-test and among several groups were tested using analysis of variance (ANOVA) with Tukey’s post-hoc test in GraphPad PRISM version 8.0 (La Jolla, CA). We performed normality tests and the compared datasets were normally distributed and had similar variability. Significant differences among groups with *p<0.05, **p<0.01; ***p<0.001 and ****p<0.0001 are indicated in the Figures, and non-significant differences are not shown. The sample size, n, indicates the total number of biological samples.

## 3. Results

### Early loss of insulin content and proinsulin accumulation in islets from insulin secretory-deficient NDM mice

**Fig 1:**
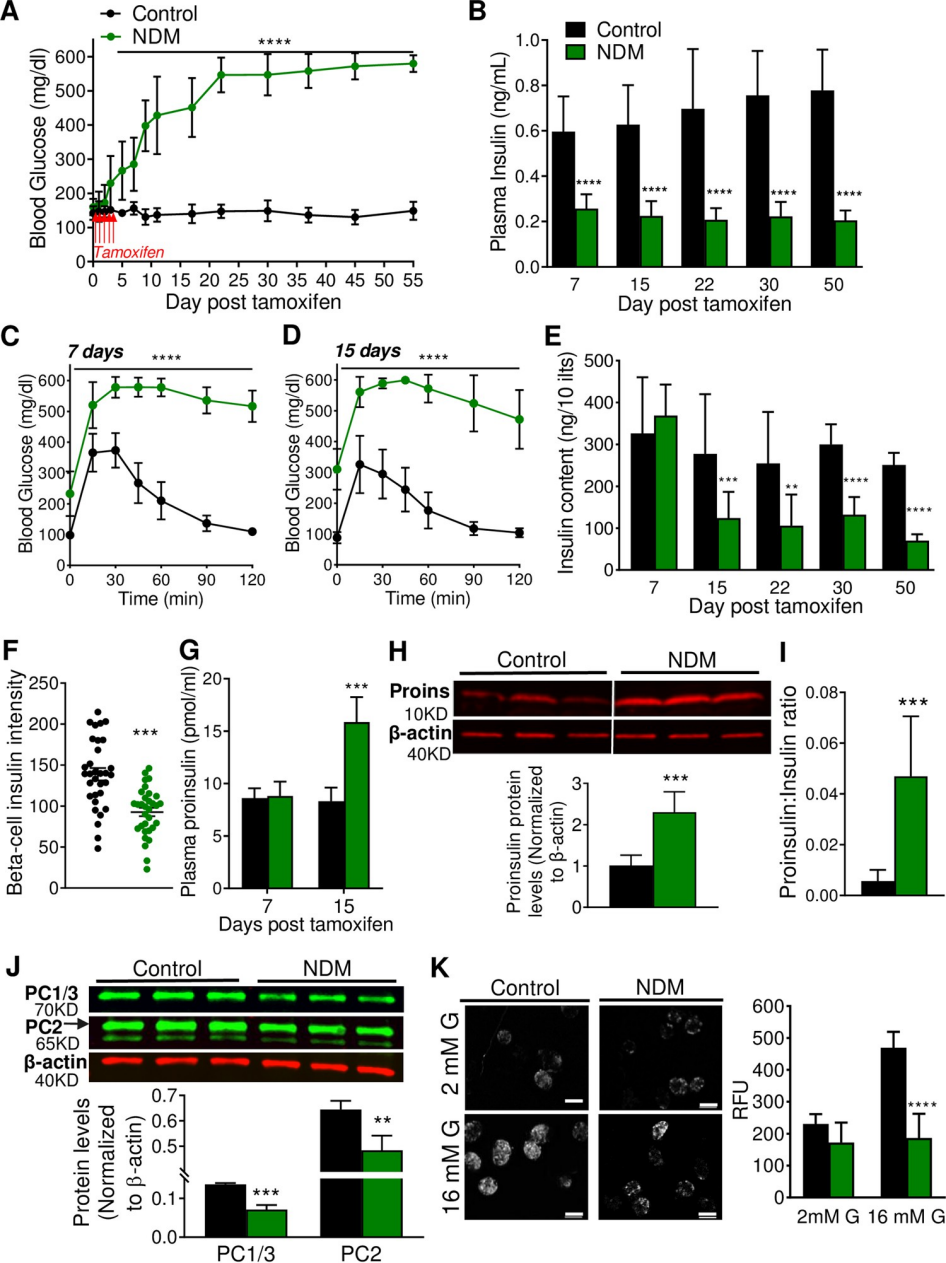
Loss of insulin content precedes loss of β-cell mass and identity in NDM (A) Non-fasting blood glucose and (B) Plasma insulin over time (n = 15–20 mice/group). Glucose tolerance test at day 7 (C) and 15 (D) post tamoxifen induction (n = 11–13 mice/group). (E) Total islet insulin content per 10 islets at different days post tamoxifen induction (n = 8–10 mice/group). (F) β-cell intensity determined in pancreatic sections immunostained with insulin (n = 8 mice/group, 3 slides from each mouse). (G) Plasma proinsulin at day 15 post tamoxifen induction (n = 7–8 mice/group). (H) Western blot analysis of proinsulin protein, representative (top) and quantification (bottom) on islets at day 15 post tamoxifen induction (n = 6 mice/group). (I) Proinsulin:insulin ratio at day 15 post tamoxifen induction (n = 10 mice/group) measured by Elisa. (J) Western blot analysis of prohormone convertases 1/3 and 2, representative (top) and quantification (bottom) on islets at day 15 post tamoxifen induction (n = 4 mice/group). (K) Calcium imaging, representative (left) and quantification (right) at 2- and 16-mM glucose at day 15 post tamoxifen induction (n = 6 mice/group, scale-bar = 100μm). Black = controls and green = NDM. Data are expressed as mean ± SD. Significant differences **P<0.01, ***P<0.001, ****P<0.0001.

As expected, upon induction of K_ATP_-GOF expression by tamoxifen, NDM mice developed severe diabetes and a marked decrease in plasma insulin levels (Fig 1A and 1B) [9]. As predicted by the ‘switch off’ of glucose-stimulated insulin secretion, NDM mice showed significantly impaired glucose tolerance (Fig 1C and 1D). Unexpectedly however, and not explained by expression of the K_ATP_ mutation, insulin content was markedly reduced (>50%) at day 15 of diabetes (Fig 1E), correlating with decreased insulin immunostaining (Fig 1F). This was accompanied by a significant increase in plasma proinsulin (Fig 1G), islet proinsulin protein levels (Fig 1H) and proinsulin:insulin content ratio (Fig 1I). Prohormone convertases 1/3 and 2, which direct the endoproteolytic cleavage of proinsulin to insulin and C-peptide, were significantly decreased in NDM islets (Fig 1J), suggesting reduced proinsulin processing as a potential cause for increased proinsulin. As expected by the presence of overactive K_ATP_-channels in β-cells, NDM islets at 15 days of diabetes did not demonstrate an increase in cytosolic calcium in response to high glucose (Fig 1K), confirming previous results in islets from long-standing diabetic NDM mice [13].

### Increased oxidative and ER stress in islets from NDM diabetic mice

**Fig 2:**
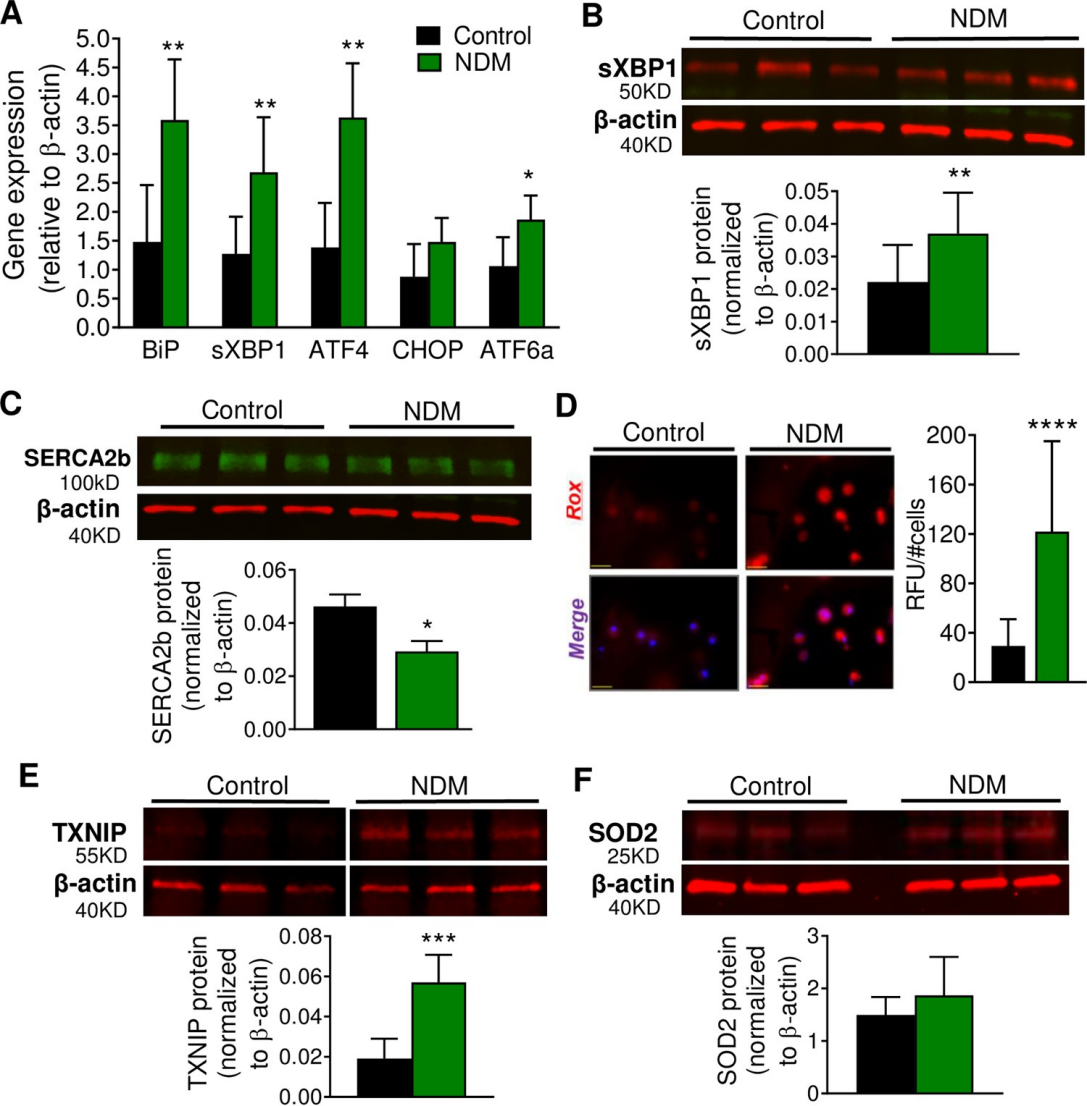
NDM islets demonstrated oxidative and ER stress in early diabetes (A) Quantitative real-time PCR analysis of gene expression of ER stress markers in islets (n = 7–9 mice/group). Western blot analysis of (B) sXBP1 and (C) SERCA2b, representative blot (top) and quantification (bottom) (n = 6 mice/group). (D) Reactive Oxygen Species (ROS) measurement in dispersed islets; representative images (left) and quantification (right) (n = 18 images from islets obtained from 3–4 mice in each group; scale bar = 25 μm). (E) Representative western blot of TXNIP (top) and quantification (bottom) (n = 6 mice/group). Samples were run on the same gel but were not contiguous. (F) Representative western blot of SOD2 (top) and quantification (bottom) (n = 6 mice/group). Black = controls and green = NDM at day 15 post tamoxifen induction. Data are expressed as mean ± SD Significant differences *P<0.05, **P<0.01, ***P<0.001, ****P<0.0001.

**Fig 3:**
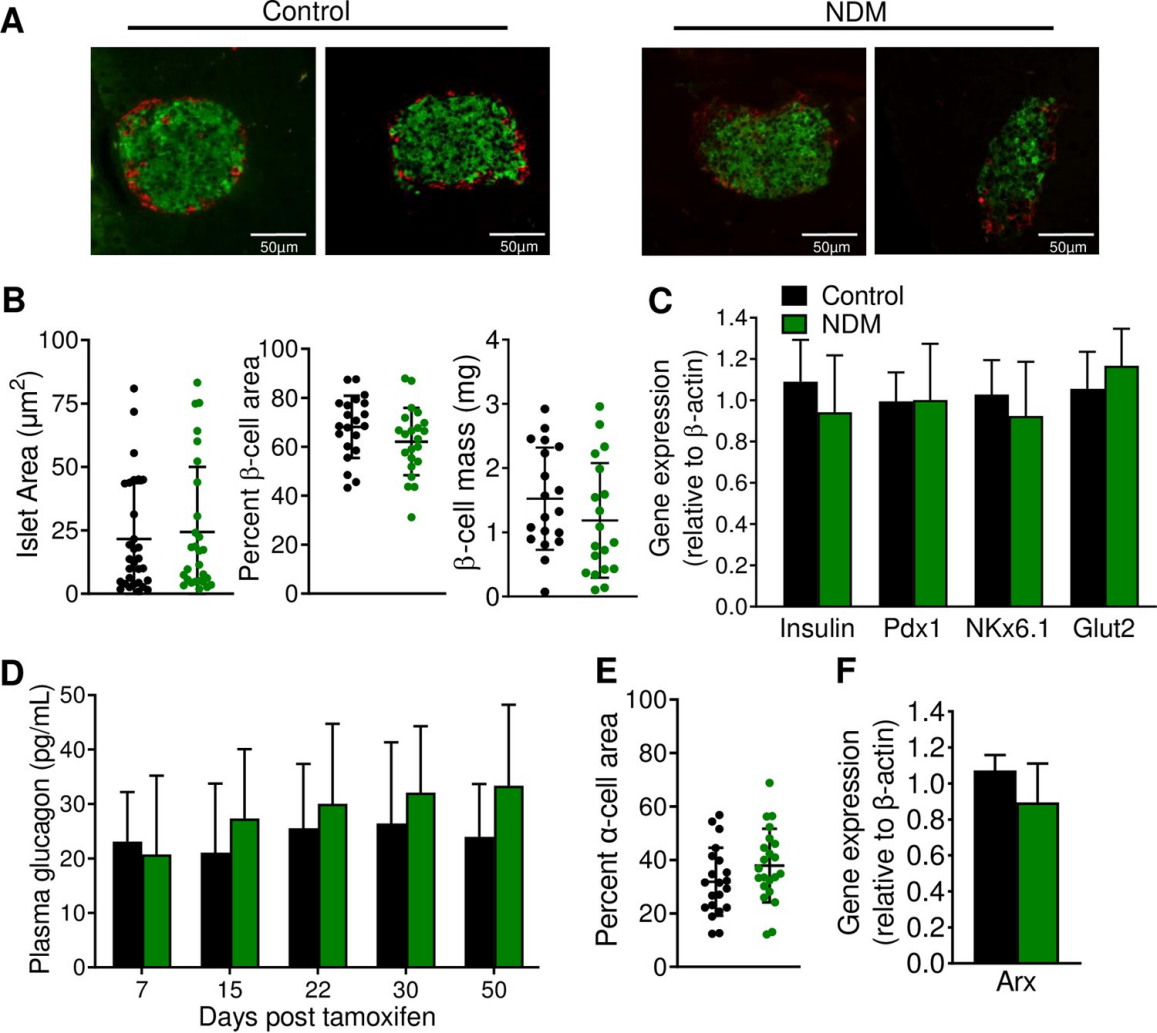
No changes in morphology or β-cell identity in islets from NDM mice at early stages of diabetes (A) Representative images of islets immunostained with insulin (green) and glucagon (red). (B) Islet area (left), Percent of β-cell area (middle) and β-cell mass (right) at day 15 of diabetes (n = 8 mice/group, 3 slides from each mouse). (C) Quantitative real-time PCR analysis of gene expression of mature β-cell identity markers in islets (n = 5–8 mice/group) at day 15 of diabetes. (D) Plasma glucagon over time during development and progression of diabetes. (E) Percent of α-cell area (n = 8 mice/group, 3 slides from each mouse), and (F) Quantitative real-time PCR analysis of gene expression of the α-cell identity marker *Arx* in islets at day 15 of diabetes (n = 4 mice/group). Black = controls and green = NDM at day 15 post-tamoxifen injection. Data are expressed as mean ± SD.

Proinsulin biosynthesis accounts for ~50% of total protein synthesis of β-cells, placing a high secretory demand on the ER where proinsulin undergoes its initial folding [34]. Accumulation of proinsulin in β-cells generally indicates activation of the ER stress pathway [35]. Islets from NDM mice demonstrated a significant increase in message RNA levels in the ER stress markers BiP, spliced XBP1 (sXBP1), Atf4 and Atf6, and no changes in Chop (Fig 2A). Protein levels of sXBP1, a product of the IRE pathway that is critical in highly secretory cells to maintain cell stress homeostasis, was significantly increased in NDM islets (Fig 2B), and ER based ATPase sarco/endoplasmic reticulum calcium pump 2 (SERCA2b) significantly reduced (Fig 2C). Reactive oxygen species (ROS) formation (Fig 2D) and thioredoxin-interacting protein (TXNIP, a pro-oxidant protein) (Fig 2E), were also markedly increased in NDM islets at day 15 of diabetes, suggesting increased oxidative stress in early stages of diabetes. Islets are particularly susceptible to oxidative damage due to low levels of antioxidants enzymes. Correlating with this, glutathione peroxidase and catalase were not detected, and superoxide dismutase 2 (SOD2) was not altered in NDM islets (Fig 2F). Interestingly, despite the decrease in insulin content at day 15 of diabetes, islet morphology, insulin and glucagon staining were not affected (Fig 3A) in NDM islets. Moreover, total islet area, percent of β-cell area and β-cell mass (Fig 3B) as well as the β-cell identity markers insulin, *Pdx1*, *Nkx6*.*1* and *Glut2* (Fig 3C) were not altered in NDM islets. In addition, plasma glucagon did not significantly change during diabetes progression (Fig 3D), and percent of α-cell area and gene expression of the α-cell marker *Arx* remained similar in NDM and littermate controls islets at day 15 of diabetes (Fig 3E and 3F).

### Dapagliflozin therapy restores insulin content in non-obese NDM mice through reduction of β-cell stress

**Fig 4:**
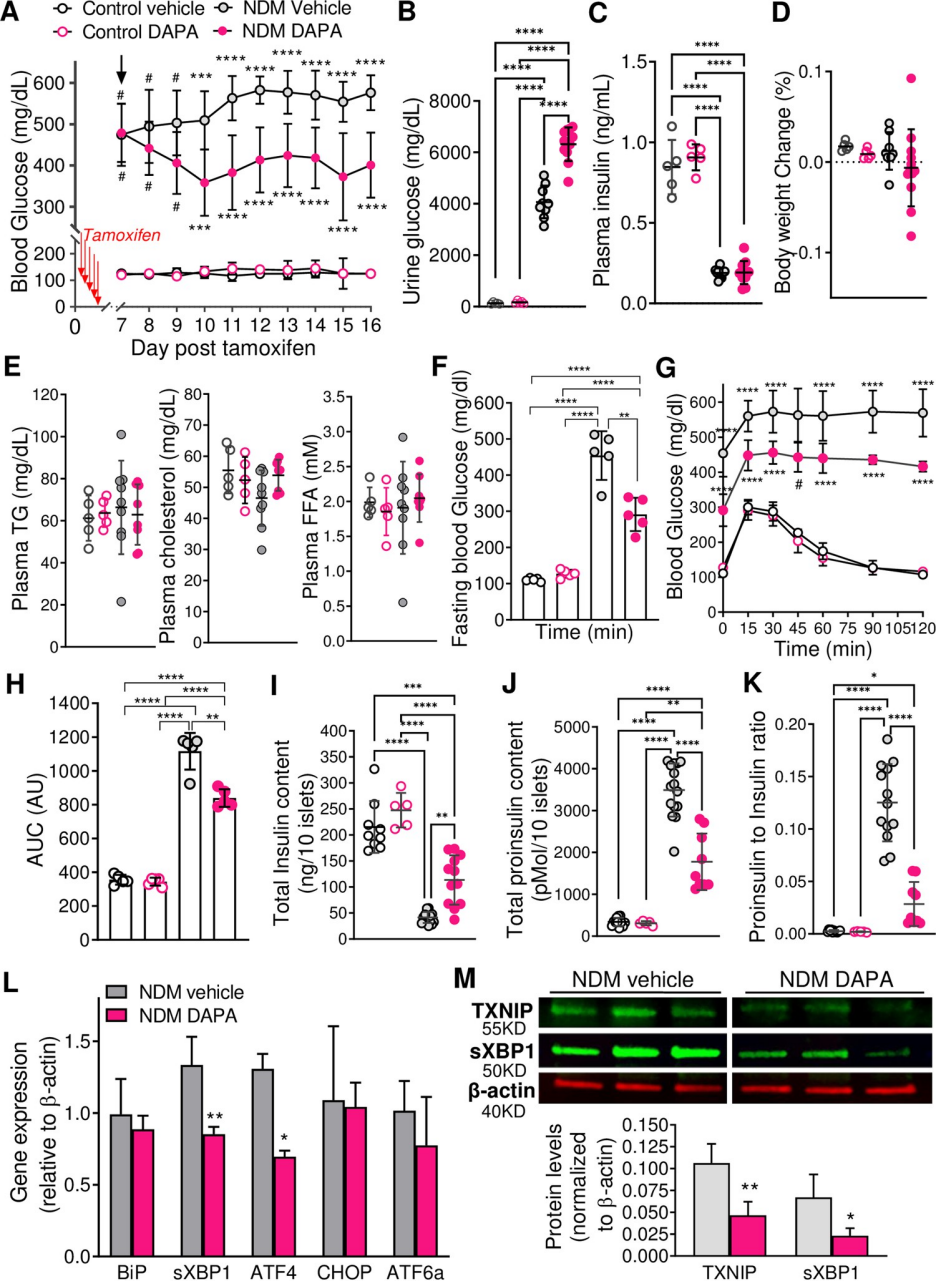
Dapagliflozin therapy in NDM mice improves β-cell function though reduction of oxidative and ER stress (A) Blood glucose in control and NDM mice administered with vehicle or dapagliflozin (DAPA) by daily oral gavage on day 7 post-tamoxifen (n = 5–15 mice/group). (B) Urine glucose (C) Plasma insulin and (D) % body weight change after 10 days of vehicle or DAPA treatment (n = 5–11 mice/group). (E) Plasma lipid panel (TG, cholesterol and FFA) (n = 5–9 mice/group). Fasting blood glucose (F), glucose tolerance test (G) and calculated area under the curve (AUC, H) from control and NDM mice after 10-day treatment with vehicle or DAPA (n = 5 mice/group). (I) Islet total insulin content, (J) Total proinsulin content and (K) Proinsulin:insulin ratio (n = 5–12 mice/group). (L) Quantitative real-time PCR analysis of gene expression of ER stress markers in islets after 10 days treatment with DAPA or vehicle (n = 4 mice/group). (M) Western blot analysis on isolated islets after 10 days treatment with DAPA or vehicle, representative blot (top) and quantification (bottom) of TXNIP and sXBP1 (n = 4–6 mice/group). Black open circles: control vehicle, pink open circles: control DAPA, grey filled circles and grey bars: NDM vehicle treated, and pink filled circles and pink bars: NDM DAPA treated mice. Arrow indicates initiation of DAPA therapy. Data are expressed as mean ± SD *P<0.05, **P<0.01, ***P<0.001, ****P<0.001 (For Fig 4A significance of ***P<0.001, ****P<0.001 respect to all other three groups, and # *p<*0.0001 respect to control vehicle and control DAPA only).

To determine whether reducing blood glucose reverts loss of insulin content and cellular stress, twelve-week old NDM mice were treated with the SGLT2i dapagliflozin (DAPA) on day 7 after tamoxifen injection. As expected, DAPA-treated NDM mice showed a significant reduction in blood glucose over time (Fig 4A), and increased urinary glucose output (Fig 4B), with no changes in plasma insulin (Fig 4C) or body weight (Fig 4D) after 10 days of DAPA therapy compared to vehicle-treated NDM. Plasma lipids such as triglycerides (TG), cholesterol, and free fatty acids (FFA) did not change by DAPA treatment (Fig 4E). DAPA-treated NDM mice showed lower fasting glucose (Fig 4F), and improved glucose tolerance (Fig 4G) with a significant reduction in the area under the curve (AUC, Fig 4H), and a mild, but not significant, improvement in insulin sensitivity (S1A Fig) compared to vehicle-treated NDM mice. Ten days of DAPA treatment was sufficient to increase insulin content (Fig 4I), reduce proinsulin (Fig 4J) and proinsulin:insulin ratio (Fig 4K), with no significant changes in the prohormone convertases PC1/3 and PC2 (S1B Fig) compared to vehicle-treated NDM mice. Importantly, islets from DAPA-treated NDM mice showed a significant decrease in sXBP1 and Atf4 message RNA levels (Fig 4L), and a marked reduction in TXNIP and sXBP1 protein levels (Fig 4M), suggesting decreased cellular stress as underlying recovery of β-cell function in NDM. No differences in any of the above-mentioned tests were observed in control mice treated with vehicle or DAPA (Fig 4A–4K). To test if alleviation of cellular stress is a unique effect of dapagliflozin or an effect of lowering blood glucose, NDM mice were implanted with low dose slow-release insulin pellets (0.1U/day/pellet) 7 days post tamoxifen, to match the timeline for DAPA therapy (S1 Text). While blood glucose was significantly reduced (S2A Fig), total islet insulin content higher (S2B Fig) and proinsulin:insulin ratio reduced (S2C Fig) in insulin-treated NDM mice compared to placebo-treated mice, cellular stress marker proteins such as TXNIP and sXBP1 were not improved (S2D Fig), suggesting unique effects of DAPA on reducing β-cell stress.

**Fig 5:**
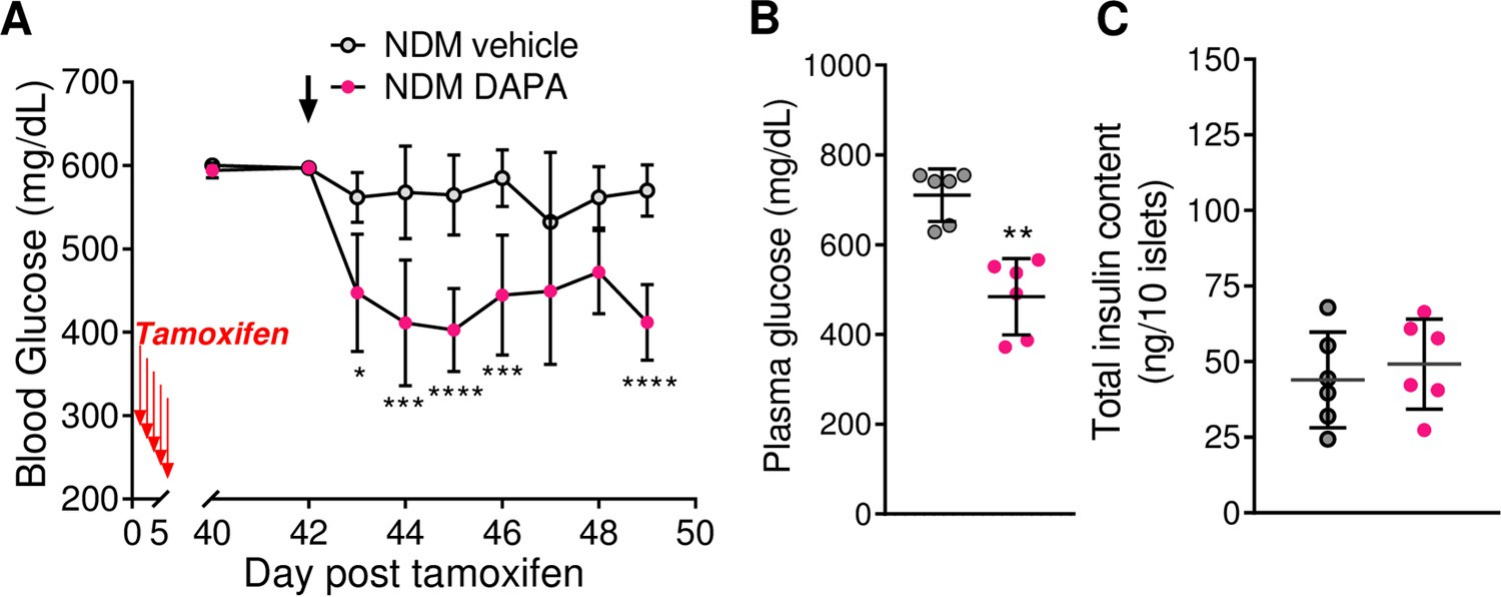
Dapagliflozin therapy does not improve insulin content in long-standing NDM NDM mice treated with vehicle or DAPA, starting on day 42 post-tamoxifen (A) Blood glucose over time and (B) plasma glucose 10 days after treatment (n = 6 mice/group). (C) Total islet insulin content 10 days after treatment with vehicle or DAPA (n = 6 mice/group). Grey = vehicle-treated NDM and pink = DAPA-treated NDM mice. Arrow indicates initiation of DAPA therapy. Data are expressed as mean ± SD *P<0.05, **P<0.01, ***P<0.001, ****P<0.001.

Notably however, despite the significant decrease in blood glucose levels over time and 10 days after treatment (Fig 5A and 5B), total insulin content (Fig 5C) was not improved in islets from NDM mice when therapy was initiated at >42 days of diabetes. These results suggest inability of SGLT2i to improve β-cell function in animals with long-standing diabetes when they had already lost β-cell mass and identity.

### Dapagliflozin therapy reduced cellular stress in mouse models of obesity and T2D

Animal models of obesity and T2D diabetes such as leptin receptor deficient *db/db* and leptin deficient *ob/ob* mice were also treated with DAPA (S1 Text). DAPA-treated *db/db* mice showed a marked reduction in blood glucose (S3A Fig), no significant changes in plasma insulin or plasma lipid levels (S3B and S3C Fig), and a significant increase in insulin content and decrease in TXNIP and sXBP1 protein levels (S3D and S3E Fig) compared to vehicle treated *db/db* mice. Similarly, *ob/ob* mice treated with DAPA showed reduction in blood glucose, although less consistent compared to the other two mouse models (S4A Fig), no significant changes in plasma insulin or plasma lipids (S4B and S4C Fig), and an increase in islet insulin content and decrease in XBP1 and TXNIP protein levels (S4D and S4E Fig) compared to vehicle-treated *ob/ob* mice.

## 4. Discussion

Loss of pancreatic β-cell mass and identity, and increased apoptosis have been demonstrated in T2D and T1D individuals [1, 3, 5, 36], and in animal models of monogenic diabetes as well as T1D and T2D diabetes [2, 4, 10, 11, 37]. Beta-cell oxidative and ER stress has been shown to be involved in β-cell failure in both T1D and T2D [36]. We demonstrated here that this is also the mechanism underlying early loss of insulin content in a mouse model of monogenic K_ATP_-GOF induced NDM in the absence of hyperglycemia-induced insulin hypersecretion and β-cell exhaustion. Decreased insulin content and prohormone convertases, and increased proinsulin and cellular stress are early events in NDM. Decreased prohormone convertases could contribute to the backlog of proinsulin to insulin conversion with proinsulin accumulation, but they could also be a consequence of diminished ER efficiency thus contributing to a vicious cycle. These early changes occurred in the absence of loss of β-cell mass and identity, which is observed in long-standing diabetes. These results are in agreement with similar mechanisms of β-cell failure and loss of functional β-cell mass that occur in humans and mouse models of both T1D and T2D/obesity [36, 38, 39], thus highlighting common execution pathways for β-cell failure in different forms of diabetes.

Although some studies demonstrated that treatment with SGLT2i preserved β-cell mass in ZDF rats [40], streptozotocin-induced T1D mice [32] and in *db/db* and *ob/ob* mouse models of obesity and T2D [25–30, 41], the underlying mechanism of this protection remains elusive. Reduced insulin content and increased intracellular proinsulin [42], as well as increased Atf4 and Chop message RNA and sXBP1 protein levels [43, 44] in islets from *db/db* mice also suggest increased ER stress in T2D/obesity. We demonstrate here for the first time that SGLT2i therapy increased islet insulin content, and decreased proinsulin levels through reduction in β-cell oxidative and ER stress in mouse models of monogenic NDM and obese/T2D. Our demonstration of lack of restoration of insulin content in NDM mice treated with DAPA at day 42 of diabetes correlate with no improvements in insulin biosynthesis/secretion in 16 week old *db/db* mice treated with luseogliflozin [25], and with absence of preservation of β-cell function in 15–20 week old *db/db* mice treated with dapagliflozin [28]. This suggests that there is a point of no return for improvement of islet function by SGLT2i, when β-cells had already lost their mature identity. These studies also correlate with those demonstrating that dapagliflozin was effective as monotherapy at early stages of human T2D, but only as add-on mediation to other antidiabetic drugs in individuals with long-standing diabetes [45].

In addition to the effect of lowering blood glucose, SGLT2i also act as insulin-sensitizing agents. While some T2D individuals showed an improvement in insulin sensitivity, reduced HbA1c and weight loss by SGLT2i therapy [46], others did not [47]. Obese *db/db* mice treated with empagliflozin [41, 48], and *db/db* mice with genetic deletion of SGLT2 [31] demonstrated improved insulin sensitivity. However, lack of significant changes in insulin sensitivity and lipid profile in 10-day DAPA-treated NDM mice suggests additional effects of SGLT2i in insulin-resistant/obese states. However, longer SGLT2i therapy could lead to a reduction in plasma lipids and therefore additional improvements in β-cell function as it has been shown in mouse models of obesity/T2D [49–51].

Increased ER stress and decreased insulin signaling might synergistically reduce β-cell function [52–54], which is supported by our data demonstrating restoration of insulin signaling and reduction of ER stress by DAPA therapy. However, insulin therapy was able to preserve insulin content and decrease proinsulin:insulin ratio in NDM mice, which is consistent with proinsulin synthesis not being regulated by insulin autocrine feedback on β-cells [54], but it was unable to reduce ER stress. Therefore, we speculate that synergistic effects might be induced by a combination of SGLT2i and insulin therapy, which could explain the beneficial effects of combination of these two agents in individuals with long-standing T2D [45]. In *db/db* mice, treatment with a combination of DPP-4 inhibitor and SGLT2 inhibitor exerted more beneficial effects on β-cell mass, function and maintenance of β-cell identity markers in early (7-week old) than the advanced (16-week old) phase of diabetes [55].

In conclusion, our study showed increased β-cell oxidative and ER stress as contributing factors that underlie early loss of insulin content in insulin secretory-deficient monogenic NDM, and that reduction of oxidative and ER stress by SGLT2i, restores insulin content and β-cell function. The mechanism underlying these effects was also demonstrated in mouse models of obesity and T2D, suggesting common pathways for recovery of functional β-cell mass in different forms of diabetes. This paradigm shift in understanding additional actions of SGLT2i correlate with the reduction of β-cell stress in NDM islets by DAPA, but not by insulin therapy. Apart from the glucose-lowering effects of SGLT2i, clinical trials revealed cardiac and renal protective effects in non-diabetic patients. The beneficial effects of SGLT2i treatment emerged from the DAPA-Heart Failure (HF) trial, in which reduction of HF and mortality was independent of reduction of hemoglobin A1C, and also from use of SGLT2i in experimental models of HF, in which cardiac improvements were independent of diabetes/hyperglycemia [56, 57]. Moreover, accumulating preclinical studies demonstrate the therapeutic benefits of SGLT2i as powerful antioxidants in human diseases [58]. Thus, we may re-conceptualize the role of SGLT2i as organ-protective agents, including the endocrine pancreas in this study, by promoting adaptive cellular reprogramming of stressed cells for survival and function [59, 60]. From a therapeutic perspective, our study strongly supports the use of SGLT2i as monotherapy in initial stages to preserve functional β-cell mass in monogenic diabetes, and as add-on combinational therapy to other antidiabetic agents in long-standing diabetes.

## Supporting information

S1 Text Animals and treatments. For insulin treatment, NDM mice were randomly separated and implanted with low-dose insulin (0.1U/day/implant, LinBit, Canada) or placebo pellets at day 7 after tamoxifen induction, as previously described [10], and blood glucose was monitored daily as in Materials and Methods. Data collection was stopped at predetermined, arbitrary time as 10 days after initiation of Insulin treatment. In addition, we tested the effect of DAPA in mouse models of obesity and type-2 diabetes. No data were excluded. Mice homozygous for the obese spontaneous mutation, Lepob (B6.Cg-Lepob/J) (https://www.jax.org/strain/000632) as well as mice for the leptin receptor mutation (BKS.Cg-Dock7m+/+ Leprdb/J) (https://www.jax.org/strain/000642) were obtained from The Jackson Laboratory. Mouse groups were assigned randomly, and the study was not blinded. Vehicle and DAPA treated mice were monitored through blood glucose measurements and blood serum collection, and then euthanized for *ex vivo* analysis. For all *in vitro* analyses, at least three independent animals were used. Cells and tissues from each animal were kept separated and analyzed individually. Data collection was stopped at predetermined, arbitrary time as 10 days after initiation of DAPA/vehicle treatment. No data were excluded.
(PDF)

S1 Fig Insulin tolerance and prohormone convertases in NDM mice treated with vehicle or DAPA (A) Insulin tolerance test from control and NDM mice after 10-day treatment with vehicle or DAPA, calculated as percentage from the initial blood glucose at 6-hrs fast (n = 5 mice/group). (B) Representative blot (top) and quantification (bottom) of prohormone convertases PC1/3 and PC2 (n = 4 mice/group). Black open circles: control vehicle, pink open circles: control DAPA, grey filled circles and grey bars: NDM vehicle treated, and pink filled circles and pink bars: NDM DAPA treated mice. Data are expressed as mean ± SD.
(PDF)

S2 Fig Insulin therapy in NDM mice reduces blood glucose but does not improve islet cellular stress (A) Blood glucose levels in NDM mice with subcutaneously implanted insulin (0.1U/day/implant) or placebo pellets for 10 days, beginning at day 7 post tamoxifen (n = 5–6 mice/group). (B) Total insulin content, (C) Proinsulin to insulin ratio (n = 6 mice/group), and (D) Western blot analysis, representative blots (left) and quantification (right) (n = 3–4 mice/group) on islets isolated from NDM mice after 10 days of insulin or placebo treatment. Grey bars and dots = placebo-treated NDM mice and purple bars and dots = Insulin-treated NDM mice. Data are expressed as mean ± SD. Significant differences *P<0.05, **P<0.01.
(PDF)

S3 Fig Dapagliflozin therapy reduced cellular stress in Db/db mice. (A) Blood glucose in *db/db* mice treated with vehicle or DAPA (n = 6 mice/group). (B) Plasma insulin and (C) Plasma lipids (triglycerides, cholesterol and FFA) in *db/db* mice after 10 days of vehicle or DAPA treatment (n = 6 mice/group). (D) Insulin content and (E) Western blot analysis, representative blot (top) and quantification (bottom), on islets from *db/db* mice 10-days DAPA or vehicle treated (n = 6 mice/group). Arrow indicates initiation of DAPA or vehicle treatment. Data presented as mean ± SD. Significant differences *p<0.05, **P<0.01, ****p<0.0001. Grey = *db/db* vehicle-treated and blue = *db/db* DAPA-treated mice.
(PDF)

S4 Fig Dapagliflozin therapy reduced cellular stress in Ob/ob mice. (A) Blood glucose in *ob/ob* mice treated with vehicle or DAPA (n = 6 mice/group). (B) Plasma insulin and (C) Plasma lipids (triglycerides, cholesterol and FFA) in *ob/ob* mice 10-days vehicle or DAPA treated (n = 6 mice/group). (D) Insulin content and (E) Western blot analysis, representative blot (top) and quantification (bottom), on islets from *ob/ob* mice 10-days after DAPA or vehicle treatment (n = 6 mice/group). Arrow indicates initiation of DAPA or vehicle treatment. Data represent mean ± SD. Significant differences *p<0.05, **P<0.01, ****p<0.0001. Grey = *ob/ob* vehicle-treated and orange = *ob/ob* DAPA-treated mice.
(PDF)

S1 Raw images (PDF)

## Abbreviations

NDM: neonatal diabetes mellitus
SGLT2: sodium glucose transporter 2
K_ATP_: ATP sensitive potassium channel
TXNIP: Thioredoxin Interacting protein
sXBP1: spliced X-box binding protein 1
ER: endoplasmic Reticulum
PC: proinsulin convertase
BiP: binding immunoglobulin protein
CHOP: C/EBP homologous protein
Pdx1: pancreas-duodenum homeobox 1
Nkx6.1: homeobox protein Nkx6.1
Glut2: glucose transporter 2
FFA: free fatty acid
DAPA: dapagliflozin
T2D: type 2 diabetes
