# Supplementary material for: SGLT2 inhibitors therapy protects glucotoxicity-induced β-cell failure in a mouse model of human KATP-induced diabetes through mitigation of oxidative and ER stress
Source: PLoS One. 2022 Feb 18;17(2):e0258054. doi: 10.1371/journal.pone.0258054 (PMC8856523; doi:10.1371/journal.pone.0258054)
Supplement: S1 Text — For insulin treatment, NDM mice were randomly separated and implanted with low-dose insulin (0.1U/day/implant, LinBit, Canada) or placebo pellets at day 7 after tamoxifen induction, as previously described [10], and blood glucose was monitored daily as in Materials and Methods. Data collection was stopped at predetermined, arbitrary time as 10 days after initiation of Insulin treatment. In addition, we tested the effect of DAPA in mouse models of obesity and type-2 diabetes. No data were excluded. Mice homozygous for the obese spontaneous mutation, Lepob (B6.Cg-Lepob/J) (https://www.jax.org/strain/000632) as well as mice for the leptin receptor mutation (BKS.Cg-Dock7m+/+ Leprdb/J) (https://www.jax.org/strain/000642) were obtained from The Jackson Laboratory. Mouse groups were assigned randomly, and the study was not blinded. Vehicle and DAPA treated mice were monitored through blood glucose measurements and blood serum collection, and then euthanized for ex vivo analysis. For all in vitro analyses, at least three independent animals were used. Cells and tissues from each animal were kept separated and analyzed individually. Data collection was stopped at predetermined, arbitrary time as 10 days after initiation of DAPA/vehicle treatment. No data were excluded. (PDF) [file pone.0258054.s001.pdf]

S1 Text. Animals and treatments. For insulin treatment, NDM mice were randomly separated and implanted with low-dose insulin (0.1U/day/implant, LinBit, Canada) or placebo pellets at day 7 after tamoxifen induction, as previously described [10], and blood glucose was monitored daily as in Materials and Methods. Data collection was stopped at predetermined, arbitrary time as 10 days after initiation of Insulin treatment. In addition, we tested the effect of DAPA in mouse models of obesity and type-2 diabetes. No data were excluded. Mice homozygous for the obese spontaneous mutation, Lepob (B6.Cg-Lepob/J) (https://www.jax.org/strain/000632) as well as mice for the leptin receptor mutation (BKS.Cg-Dock7m+/+ Leprdb/J) (https://www.jax.org/strain/000642) were obtained from The Jackson Laboratory. Mouse groups were assigned randomly, and the study was not blinded. Vehicle and DAPA treated mice were monitored through blood glucose measurements and blood serum collection, and then euthanized for *ex vivo* analysis. For all *in vitro* analyses, at least three independent animals were used. Cells and tissues from each animal were kept separated and analyzed individually. Data collection was stopped at predetermined, arbitrary time as 10 days after initiation of DAPA/vehicle treatment. No data were excluded.
