# Supplementary figures and images for: SGLT2 inhibitors therapy protects glucotoxicity-induced β-cell failure in a mouse model of human KATP-induced diabetes through mitigation of oxidative and ER stress
Source: PLoS One. 2022 Feb 18;17(2):e0258054. doi: 10.1371/journal.pone.0258054 (PMC8856523; doi:10.1371/journal.pone.0258054)

S1 Fig.

**A**

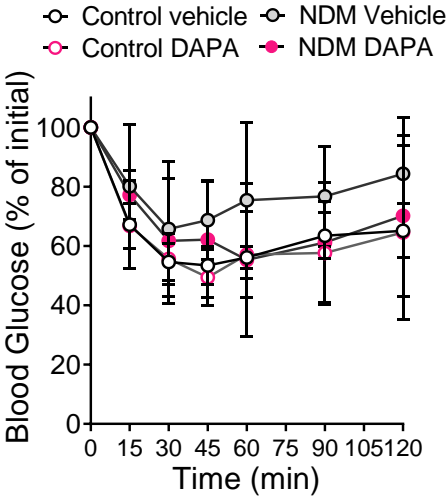

**B**

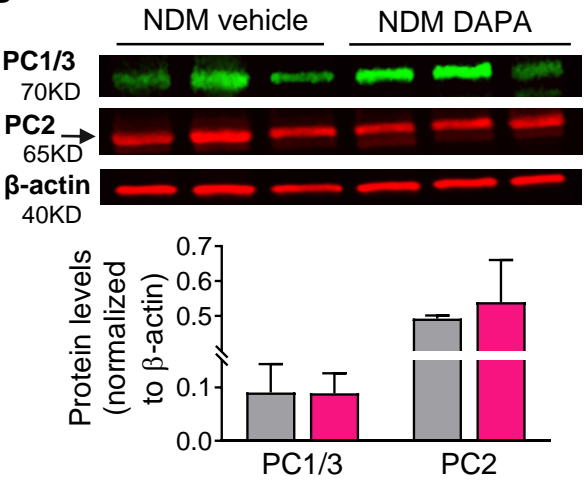

Supplement: S1 Fig — Insulin tolerance and prohormone convertases in NDM mice treated with vehicle or DAPA (A) Insulin tolerance test from control and NDM mice after 10-day treatment with vehicle or DAPA, calculated as percentage from the initial blood glucose at 6-hrs fast (n = 5 mice/group). (B) Representative blot (top) and quantification (bottom) of prohormone convertases PC1/3 and PC2 (n = 4 mice/group). Black open circles: control vehicle, pink open circles: control DAPA, grey filled circles and grey bars: NDM vehicle treated, and pink filled circles and pink bars: NDM DAPA treated mice. Data are expressed as mean ± SD. (PDF) [file pone.0258054.s002.pdf]

S2 Fig.

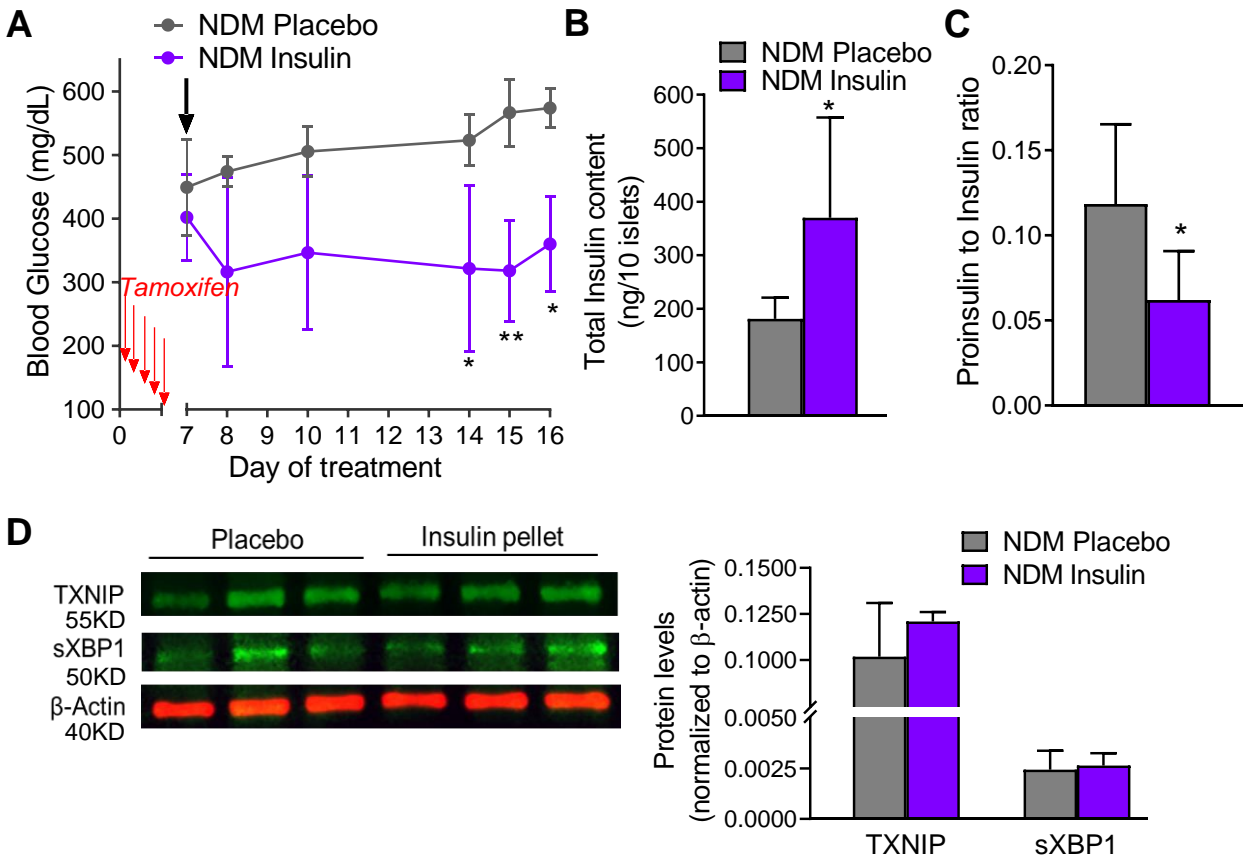

Supplement: S2 Fig — Insulin therapy in NDM mice reduces blood glucose but does not improve islet cellular stress (A) Blood glucose levels in NDM mice with subcutaneously implanted insulin (0.1U/day/implant) or placebo pellets for 10 days, beginning at day 7 post tamoxifen (n = 5–6 mice/group). (B) Total insulin content, (C) Proinsulin to insulin ratio (n = 6 mice/group), and (D) Western blot analysis, representative blots (left) and quantification (right) (n = 3–4 mice/group) on islets isolated from NDM mice after 10 days of insulin or placebo treatment. Grey bars and dots = placebo-treated NDM mice and purple bars and dots = Insulin-treated NDM mice. Data are expressed as mean ± SD. Significant differences *P<0.05, **P<0.01. (PDF) [file pone.0258054.s003.pdf]

S3 Fig.

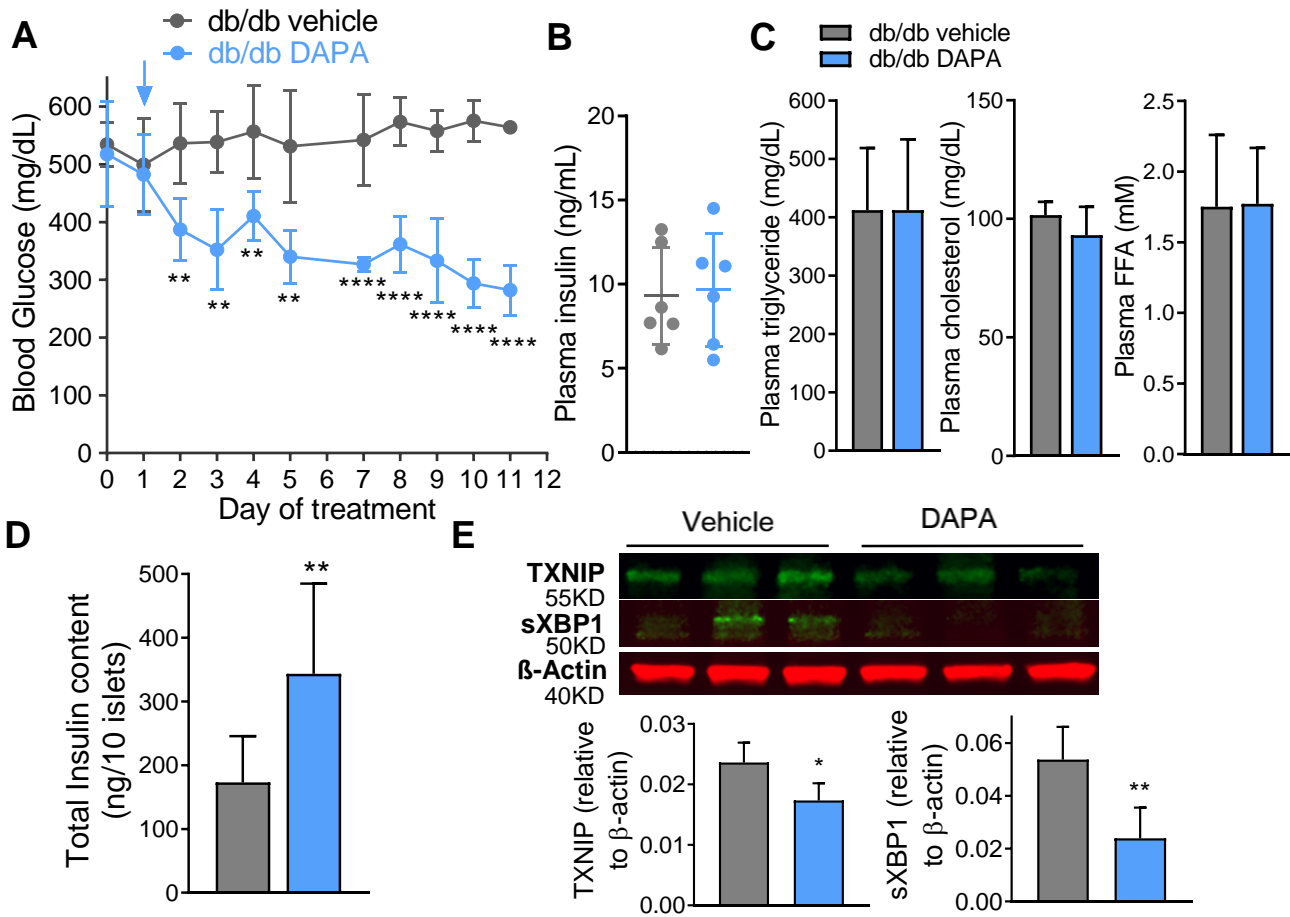

Supplement: S3 Fig — (A) Blood glucose in db/db mice treated with vehicle or DAPA (n = 6 mice/group). (B) Plasma insulin and (C) Plasma lipids (triglycerides, cholesterol and FFA) in db/db mice after 10 days of vehicle or DAPA treatment (n = 6 mice/group). (D) Insulin content and (E) Western blot analysis, representative blot (top) and quantification (bottom), on islets from db/db mice 10-days DAPA or vehicle treated (n = 6 mice/group). Arrow indicates initiation of DAPA or vehicle treatment. Data presented as mean ± SD. Significant differences *p<0.05, **P<0.01, ****p<0.0001. Grey = db/db vehicle-treated and blue = db/db DAPA-treated mice. (PDF) [file pone.0258054.s004.pdf]

S4 Fig.

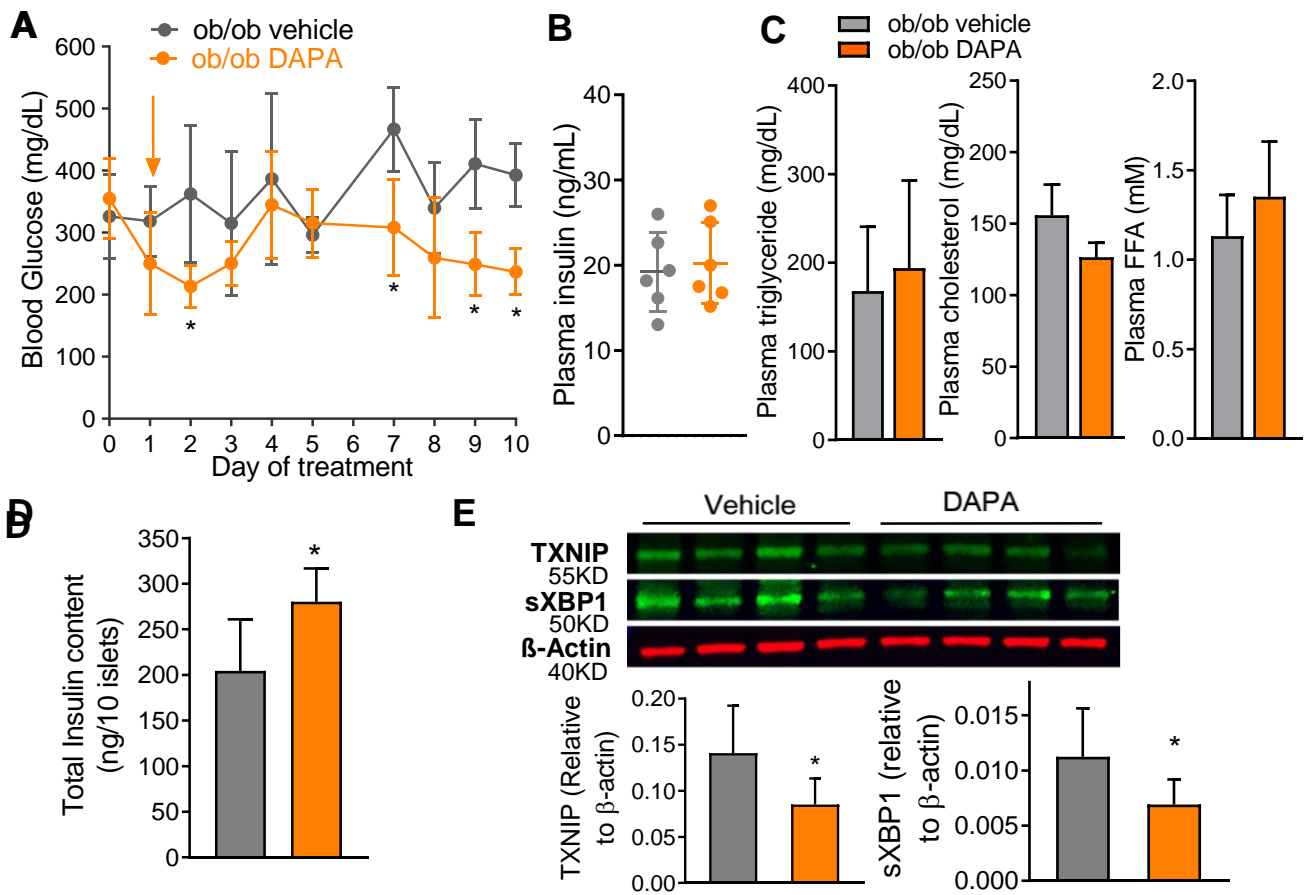

Supplement: S4 Fig — (A) Blood glucose in ob/ob mice treated with vehicle or DAPA (n = 6 mice/group). (B) Plasma insulin and (C) Plasma lipids (triglycerides, cholesterol and FFA) in ob/ob mice 10-days vehicle or DAPA treated (n = 6 mice/group). (D) Insulin content and (E) Western blot analysis, representative blot (top) and quantification (bottom), on islets from ob/ob mice 10-days after DAPA or vehicle treatment (n = 6 mice/group). Arrow indicates initiation of DAPA or vehicle treatment. Data represent mean ± SD. Significant differences *p<0.05, **P<0.01, ****p<0.0001. Grey = ob/ob vehicle-treated and orange = ob/ob DAPA-treated mice. (PDF) [file pone.0258054.s005.pdf]
