# Supplementary material for: SGLT2 inhibitors therapy protects glucotoxicity-induced β-cell failure in a mouse model of human KATP-induced diabetes through mitigation of oxidative and ER stress
Source: PLoS One. 2022 Feb 18;17(2):e0258054. doi: 10.1371/journal.pone.0258054 (PMC8856523; doi:10.1371/journal.pone.0258054)

Figure 1 H- Top: Proinsulin and beta-actin. Yellow boxes designate images used in manuscript (middle lanes of actin did not transfer well and therefore were NOT used for quantification). **Bottom:** Cyclophilin B was also used as a housekeeping gene to ensure even loading.

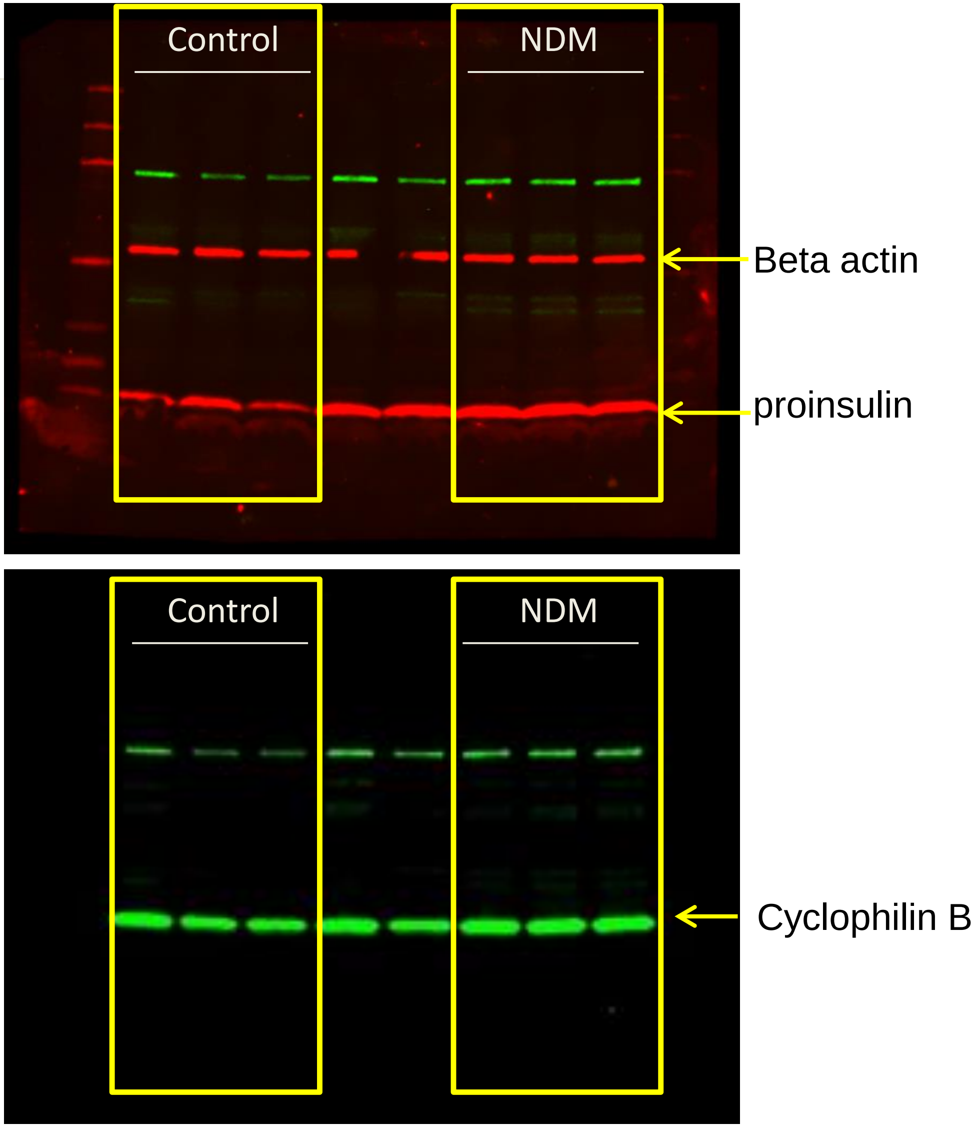

Figure 1 J- Top: Prohormone convertase-2, **Bottom**: Prohormone convertase-1/3

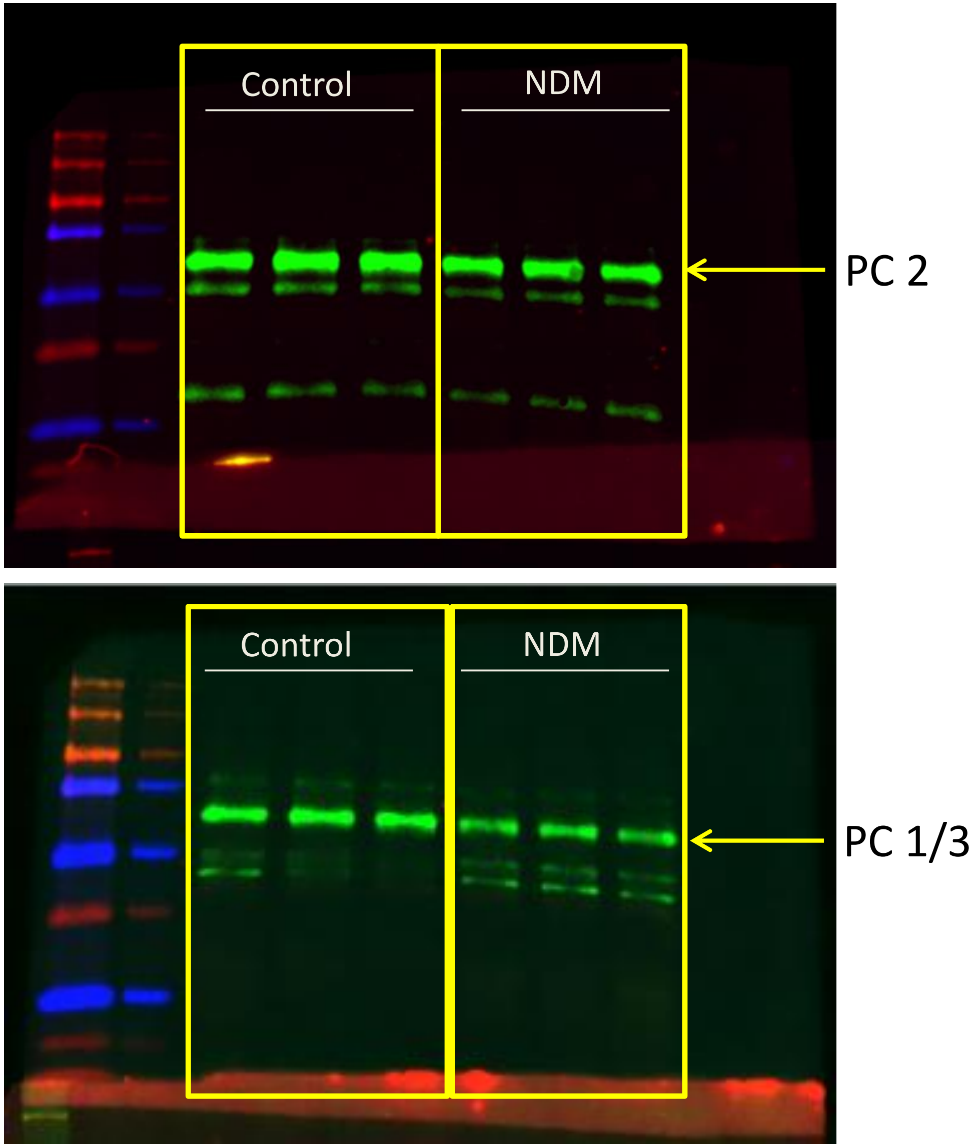

Figure 2B and 2C- sXBP1 and SERCA

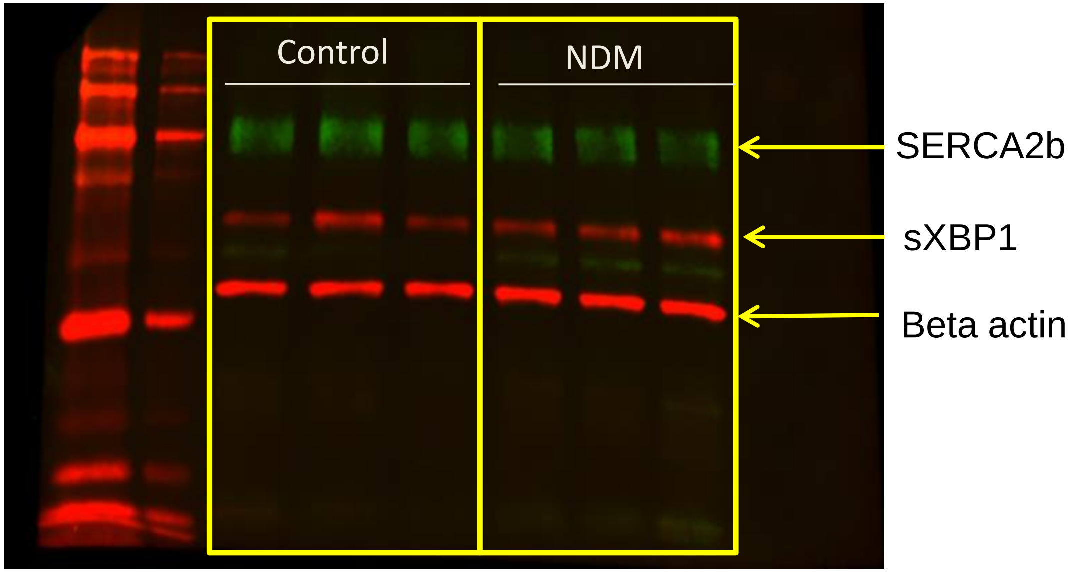

Figure 2E- TXNIP.

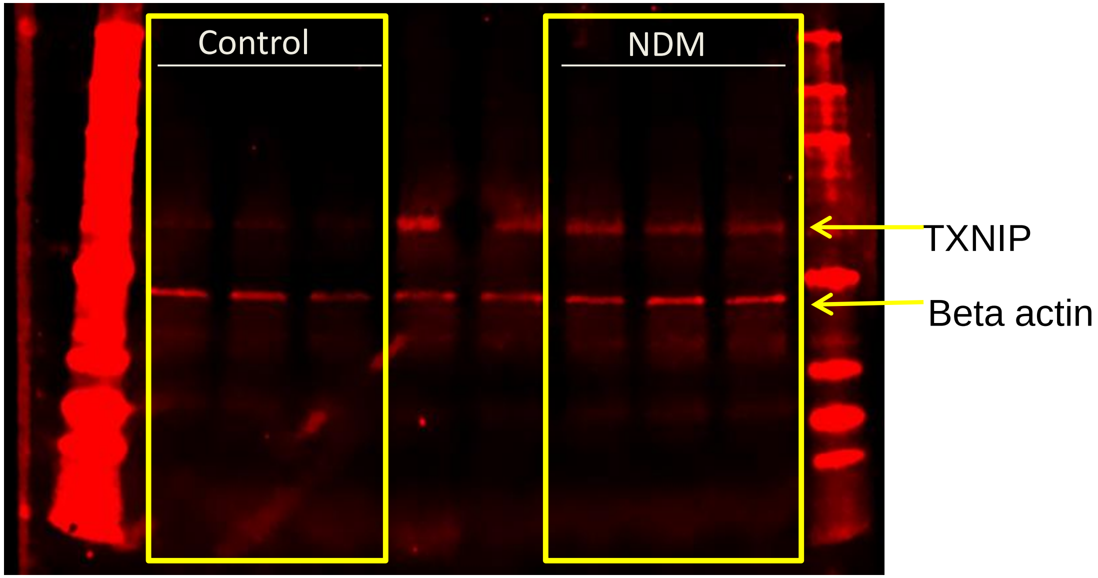

Yellow boxes designate images used in manuscript. Since middle TXNIP lanes had a clear visible air pocket, those samples were NOT used for quantification.

Figure 2F- SOD 2

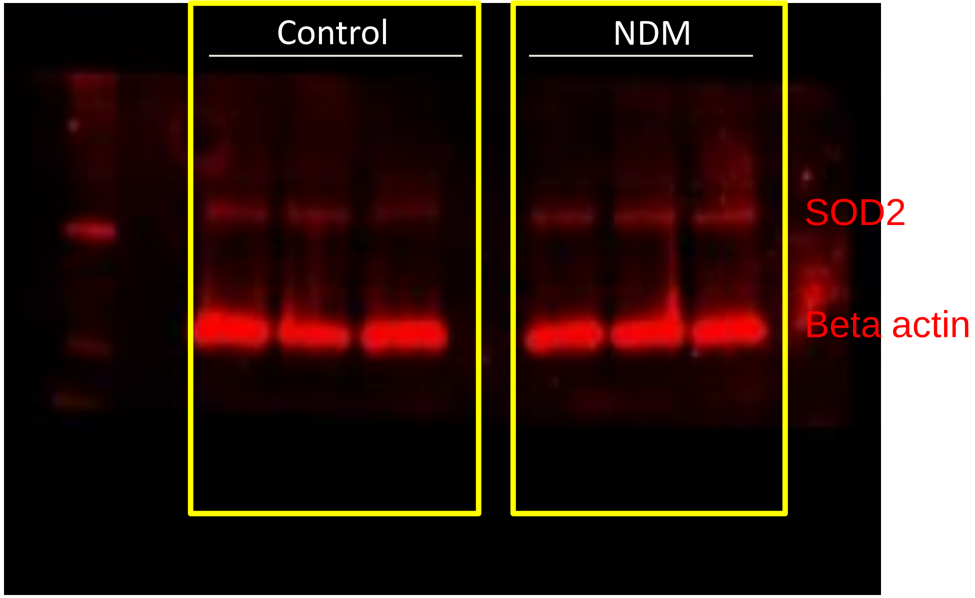

Figure 4M- islets from NDM mice, vehicle or DAPA treated. **Top:** sXBP1 and **Bottom:** TXNIP

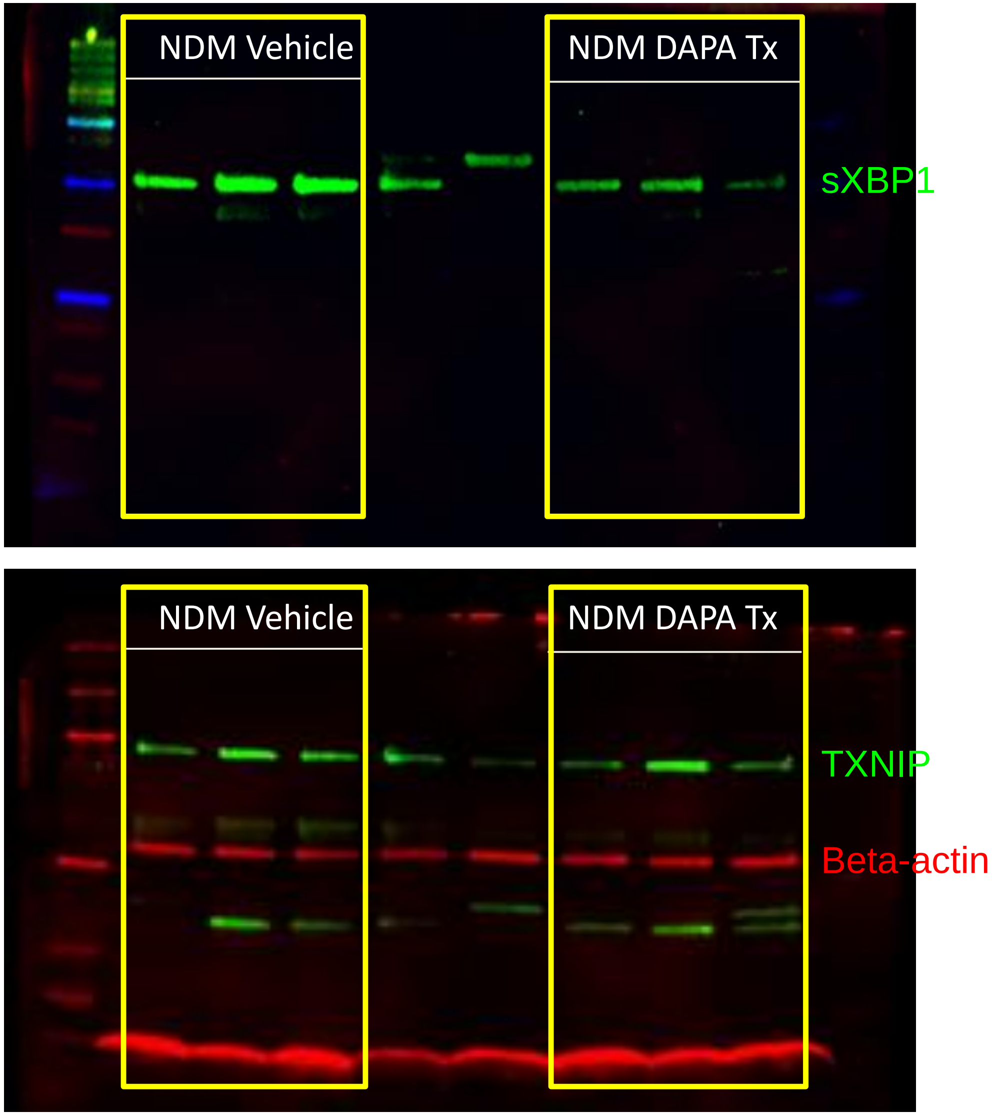

Figure S1B - Islets from NDM mice, vehicle or DAPA treated. **Top:** PC1/3 and **Bottom:** PC2

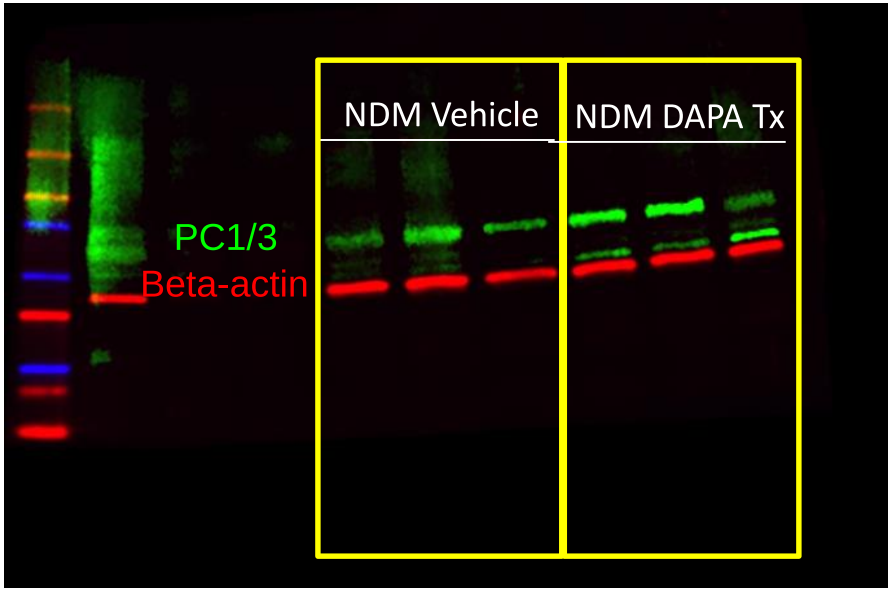

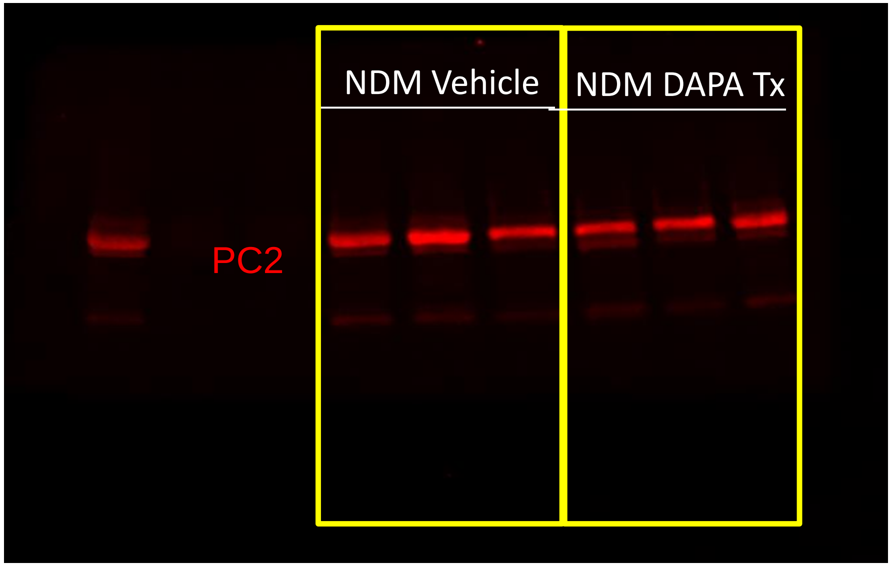

Figure S2D - Islets from control and NDM mice treated with insulin. **Top**: TXNIP and **Bottom**: sXBP1

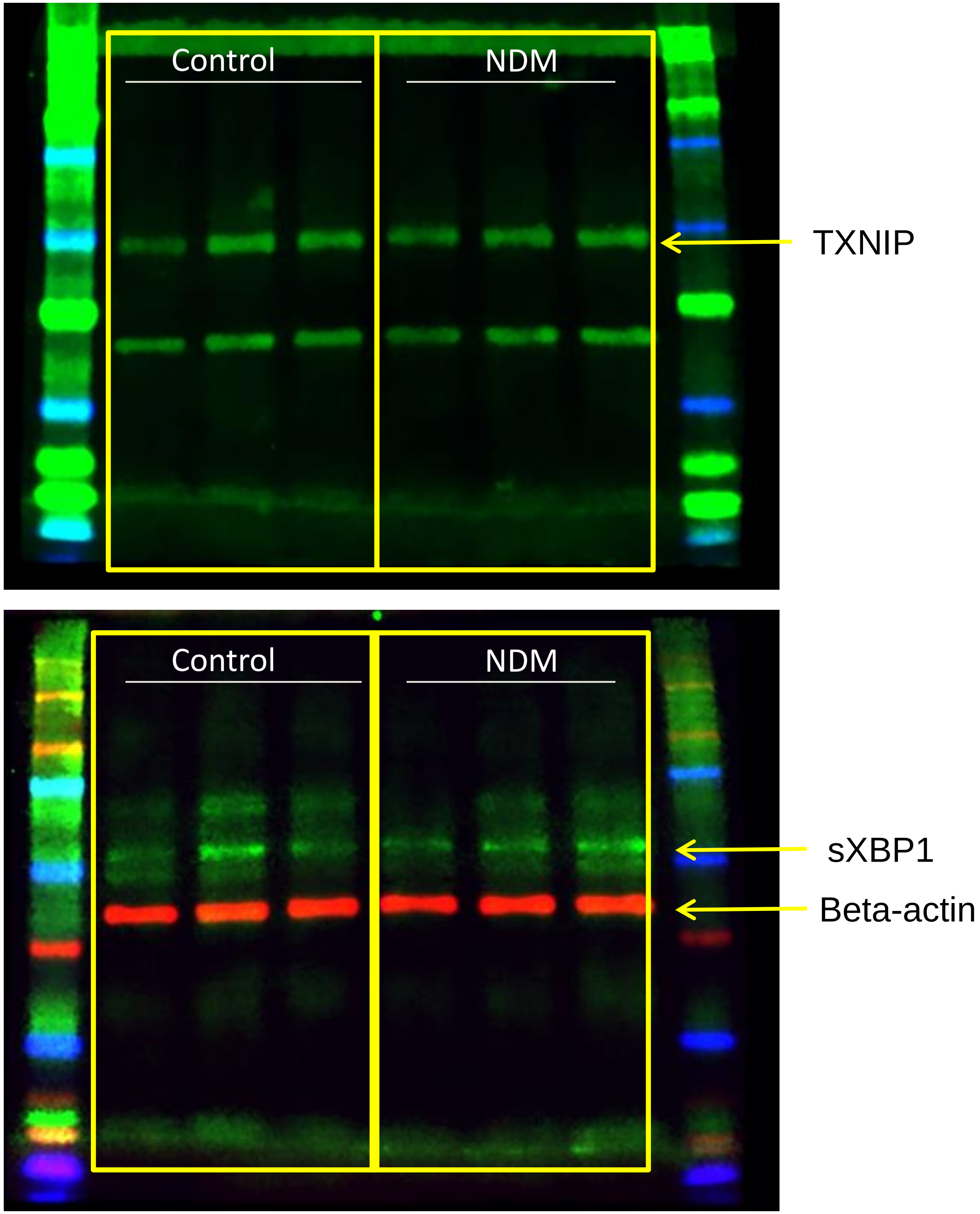

Figure S3E: Islets from db/db mice, vehicle or DAPA treated. Top: sXBP1 and Bottom: TXNIP

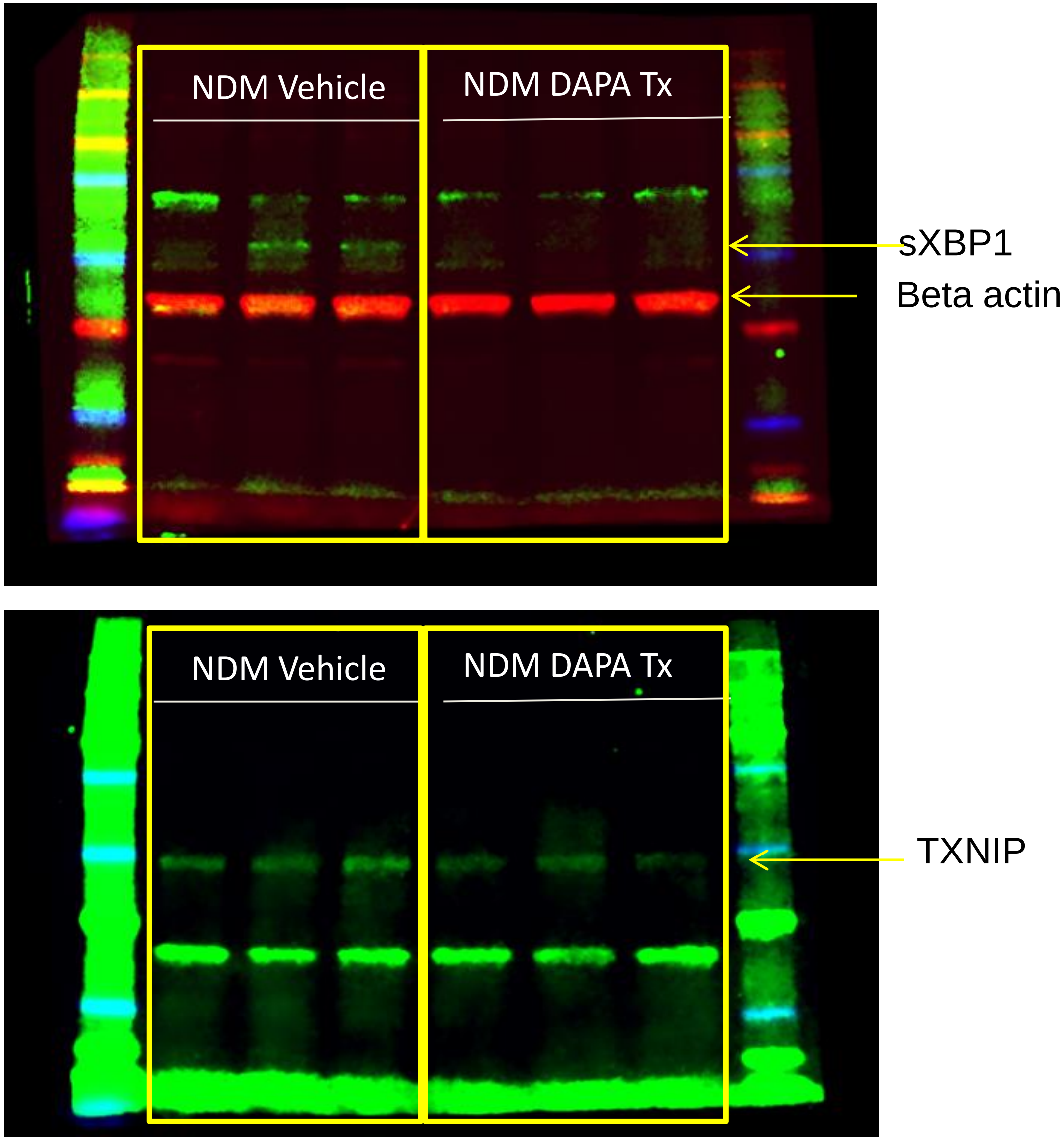

Figure S3J: islets from OB/OB mice, vehicle or DAPA treated. **Top:** sXBP1 and **Bottom:** TXNIP

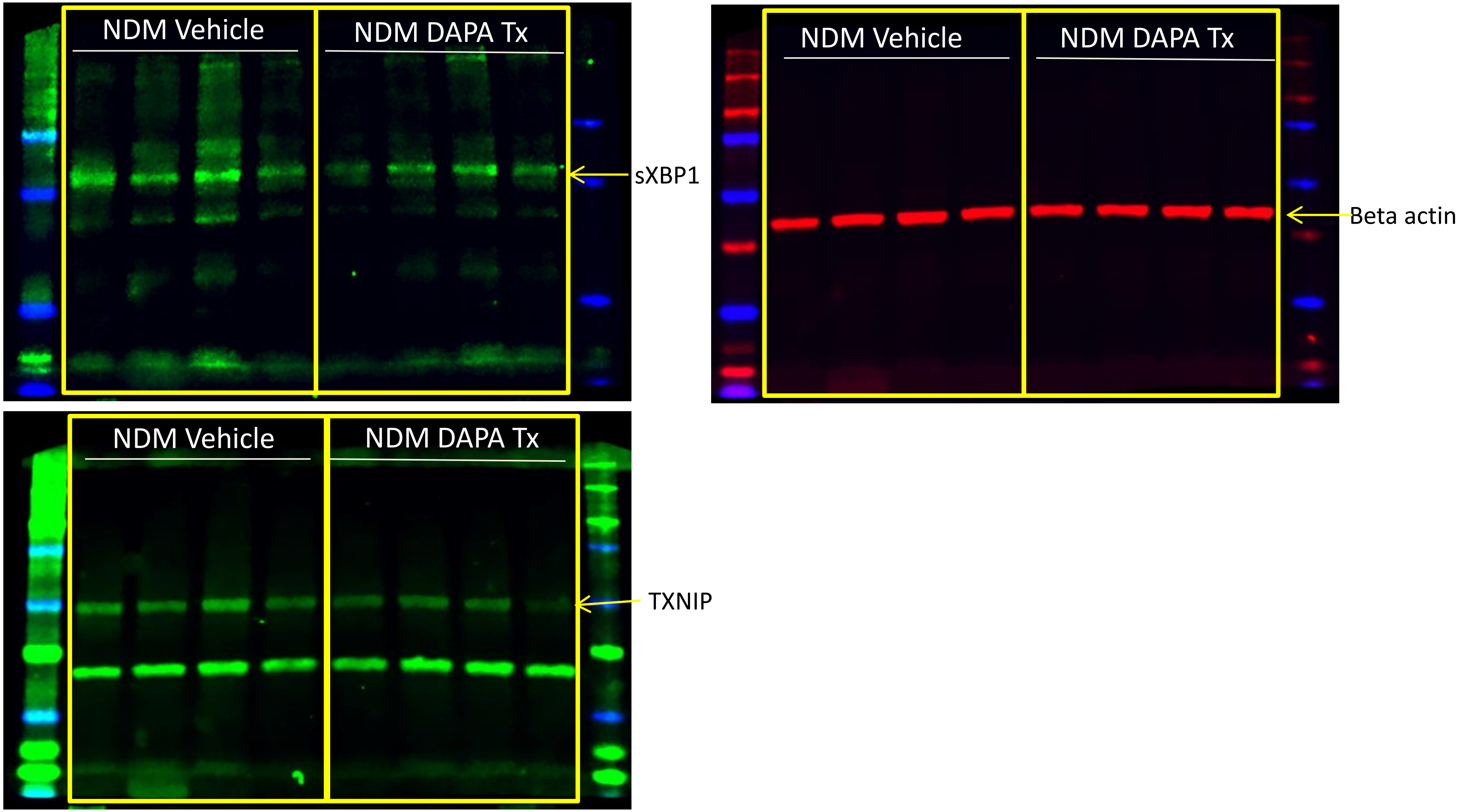

Supplement: S1 Raw images — (PDF) [file pone.0258054.s006.pdf]
